# Performance Validity Test Failure in the Clinical Population: A Systematic Review and Meta-Analysis of Prevalence Rates

**DOI:** 10.1007/s11065-023-09582-7

**Published:** 2023-03-06

**Authors:** Jeroen J. Roor, Maarten J. V. Peters, Brechje Dandachi-FitzGerald, Rudolf W. H. M. Ponds

**Affiliations:** 1 Department of Medical Psychology, VieCuri Medical Center, Venlo, The Netherlands; 2 School for Mental Health and Neuroscience, Maastricht University, Maastricht, The Netherlands; 3 Department of Clinical Psychological Science, Faculty of Psychology and Neuroscience, Maastricht University, Maastricht, The Netherlands; 4 Department of Medical Psychology, Amsterdam University Medical Centres, location VU, Amsterdam, The Netherlands; 5 Faculty of Psychology, Open University, Heerlen, The Netherlands

**Keywords:** Prevalence, Base rate, Performance validity test, Invalid performance, Meta-analysis, Clinical assessments

## Abstract

Performance validity tests (PVTs) are used to measure the validity of the obtained neuropsychological test data. However, when an individual fails a PVT, the likelihood that failure truly reflects invalid performance (i.e., the positive predictive value) depends on the base rate in the context in which the assessment takes place. Therefore, accurate base rate information is needed to guide interpretation of PVT performance. This systematic review and meta-analysis examined the base rate of PVT failure in the clinical population (PROSPERO number: CRD42020164128). PubMed/MEDLINE, Web of Science, and PsychINFO were searched to identify articles published up to November 5, 2021. Main eligibility criteria were a clinical evaluation context and utilization of stand-alone and well-validated PVTs. Of the 457 articles scrutinized for eligibility, 47 were selected for systematic review and meta-analyses. Pooled base rate of PVT failure for all included studies was 16%, 95% CI [14, 19]. High heterogeneity existed among these studies (Cochran's Q = 697.97, *p* < .001; *I*^2^ = 91%; τ^2^ = 0.08). Subgroup analysis indicated that pooled PVT failure rates varied across clinical context, presence of external incentives, clinical diagnosis, and utilized PVT. Our findings can be used for calculating clinically applied statistics (i.e., positive and negative predictive values, and likelihood ratios) to increase the diagnostic accuracy of performance validity determination in clinical evaluation. Future research is necessary with more detailed recruitment procedures and sample descriptions to further improve the accuracy of the base rate of PVT failure in clinical practice.

**Supplementary Information:**

The online version contains supplementary material available at 10.1007/s11065-023-09582-7.

## Introduction

Neuropsychological assessment guides diagnostics and treatment in a wide range of clinical conditions (e.g., traumatic brain injury, epilepsy, functional neurological disorder, attention deficit hyperactivity disorder, multiple sclerosis, or mild cognitive impairment). Therefore, it is important that neuropsychological test results accurately represent a patients’ actual cognitive abilities. However, personal factors such as a lack of task engagement or malingering can invalidate a patient’s test performance (Schroeder & Martin, 2022). When invalid performance is not properly identified, clinicians risk attributing abnormally low scores to cognitive impairment, potentially leading to misdiagnosis and ineffective or even harmful treatments (e.g., Roor et al., 2016; van der Heide et al., 2020). Consequently, performance invalidity is not only relevant to diagnostics, but also extends to treatment efficacy (Roor et al., 2022).

Various tests are available for determining invalid performance on cognitive tests (for an overview, *see* Soble et al., 2022). Performance validity tests (PVTs) can be specifically designed to measure performance validity (i.e., stand-alone PVTs), or empirically derived from standard cognitive tests (i.e., embedded indicators). Overall, the psychometric properties of stand-alone PVTs have been found to be superior in comparison to embedded PVTs (Miele et al., 2012; Soble et al., 2022). Using well-researched stand-alone PVTs, the meta-analyses of Sollman and Berry (2011) found their aggregated mean specificity to be 0.90, with a mean sensitivity of 0.69. This finding is typical for stand-alone PVTs, for which empirical cutoff scores are chosen at a specificity of ≥ 0.90 to minimize the misclassification of a valid cognitive test performance as non-valid (i.e., a maximum 10% false positive rate).

Importantly, sensitivity and specificity should never be interpreted in isolation from other clinical metrics like base rates (Lange & Lippa, 2017). To determine the positive and negative predictive value of a PVT score, the base rate of the condition (here: performance invalidity) needs to be considered (Richards et al., 2015). Using Bayes' rule, the likelihood that PVT failure is indeed indicative of performance invalidity can be calculated based upon: 1) the base rate of invalid performance in the specific population of that individual; 2) the score of a PVT; 3) the sensitivity; and 4) the specificity of the utilized PVT (Dandachi-FitzGerald & Martin, 2022; Tiemens et al., 2020). Ignoring Bayes' rule potentially leads to overdiagnosis of invalid performance when the base rate of invalidity is low and to underdiagnosis when the base rate is high. Therefore, it is essential that base rate information is available for each PVT in a specific clinical context and, ideally, for specific clinical patient groups (Schroeder et al., 2021a).

Early surveys amongst non-forensic clinical neuropsychologists reported an expectation that only 8% of general clinical referrals would produce invalid test results (Mittenberg et al., 2002). Over the last two decades, research on validity issues in clinical practice increased significantly, and neuropsychologists have become more aware of the need to identify invalid test performance (Merten & Dandachi-FitzGerald, 2022; Sweet et al., 2021). These factors probably contributed to the findings of a nearly double median reported base rate of 15% across clinical contexts and settings in a more recent survey (Martin & Schroeder, 2020). However, there has been a delay in research examining empirically derived bases rates of invalidity in clinical settings.

To address this issue, McWhirter et al. (2020) undertook a systematic review to examine PVT failure in clinical populations. Their main finding was that PVT failure rates were common, exceeding 25% for some PVTs and clinical groups. However, their study has been criticized on several aspects. First, Kemp and Kapur (2020) did not distinguish between stand-alone and (psychometrically inferior) embedded PVTs. Second, McWhirter et al. (2020) included studies that examined PVT failure in patients with dementia and intellectual disabilities, two groups in which PVTs are strongly discouraged due to unacceptable high false-positive rates when using the standard cutoffs (Larrabee et al., 2020; Lippa, 2018; Merten et al., 2007). Third, studies with ≥ 50% of the patient sample was involved in litigation or seeking welfare benefits were excluded, other types and lower rates of external gain incentives were not characterized. Therefore, external incentives that increase PVT failure rates in patients engaged in standard clinical evaluations (Schroeder et al., 2021b) may have contributed to their reported PVT failure rates. Importantly, McWhirter et al. (2020) summarized data on PVT failure based upon the literature search and data extraction performed by one author, without considering the quality of included studies or calculating a weighted average to get a more precise estimate.

The current meta-analysis is designed to address these gaps to improve the quality of reported PVT failure findings in clinical patient groups. The main aim of the present study is to provide comprehensive information regarding the base rate of PVT failure to facilitate its interpretation in clinical practice. We calculated pooled estimates of the base rate of PVT failure across the type of clinical context, distinct clinical patient groups, the potential for external incentives, and per PVT.

## Methods

### Search Strategy

This meta-analysis was conducted in accordance with updated Preferred Reporting Items for Systematic Review and Meta-analyses guidelines (PRISMA; Page et al., 2021). A review protocol was registered at inception on PROSPERO (ID: CRD42020164128). The protocol was slightly modified to further improve the quality of included studies. Specifically, only stand-alone PVTs were included that met the restrictive selection criteria per Sollman and Berry (2011), and one additional database was searched. Electronic databases (PubMed/MEDLINE, Web of Science, and PsychINFO) were comprehensively searched using multiple terms for performance validity and neuropsychological assessment (*see* Online Resource 1 for detailed search strategies). Finally, we chose to focus solely on the base rate of PVT failure without also addressing its impact on treatment outcome. The final search was conducted on November 5, 2021.

### Study Selection

All studies in this systematic review and meta-analysis were performed in a clinical evaluation context of adult patients (18 + years of age), using standard/per manual administration procedure and cutoffs for the five stand-alone performance validity tests (PVTs) from Sollman and Berry (2011). These five PVTs are: the Word Memory Test (WMT; Green, 2003), the Medical Symptom Validity Test (MSVT; Green, 2004), the Test of Memory Malingering (TOMM; Tombaugh, 1996), the Victoria Symptom Validity Test (VSVT; Slick et al., 1997), and the Letter Memory Test (LMT; Inman et al., 1998). Based upon Grote et al. (2000), a higher cutoff was used for the hard items of the VSVT in patients with medically intractable epilepsy. All studies were original, peer-reviewed, and published in English. Studies were excluded if they examined PVT failure rate in a non-clinical context (i.e., forensic/medico-legal context, data generated for research purposes). Studies that only addressed PVT failure in a sample already selected upon initially passing/failing a PVT were equally excluded (typically known-groups design). Studies performed on patients diagnosed with intellectual disability or dementia were excluded, as well as studies with a small (sub)sample size (*N* < 20). Finally, we chose to exclude studies of Veterans/military personnel since the distinction between clinical and forensic evaluations are difficult to make within the context of the Veterans Affairs (VA) system (Armistead-Jehle & Buican, 2012).

Unique patient samples were ensured by carefully screening for similar samples used in different studies. In case multiple studies examined the same patient sample, data with the largest sample size was included, or, when equal, the most recent paper.

### Data Collection and Extraction

References resulting from the searches in PubMed/MEDLINE, Web of Science, and PsychINFO were imported into a reference manager (EndNote X8). After automatic duplicate removal, one of the investigators (JR) manually removed the remaining duplicate references. First, a single rater (JR) screened all titles and abstracts for broad suitability and eligibility. Doubtful references were addressed with a second rater (MP). If doubts remained, references were included for full-text scrutinization. Second, two independent raters (MP and JR) reviewed the remaining full-texts based on the mentioned inclusion and exclusion criteria, for which the online systematic review tool Rayyan (Ouzzani et al., 2016) was used. The interrater reliability was substantial (Cohen’s *k* = 0.63), and agreement 89.83%. A sizable number of studies failed to clearly state information used for inclusion in the current study, which contributed to the suboptimal agreement between the two independent raters. Therefore, corresponding authors were contacted when additional information was required (e.g., regarding clinical context, utilized PVT cutoff, number of subjects that were provided and failed a PVT, or language/version of the utilized PVT). Non-responders were reminded twice, and if no author response was elicited, studies were excluded. Discrepancies were resolved by discussion with a third and fourth reviewer (BD and RP). Finally, one investigator (JR) extracted relevant information from the included full-text articles, such as setting, sample size, mean age, and utilized PVT(s) according to a standardized data collection form (*see* Online Resource 2).

### Statistical Analyses

Statistical analysis was performed using MetaXL version 5.3 (www.epigear.com), a freely available add-in for meta-analysis in Microsoft Excel. Independence of effect sizes, a critical assumption in random-effects meta-analyses, was examined by checking if and how many studies used multiple, potentially inter-correlated PVTs from the same patient sample (Cheung, 2019). The frequency of PVT failure from the individual studies were pooled into the meta-analysis using a double-arcsine transformation. Back transformation was performed to report the pooled prevalence rates. We chose to use this transformation method to stabilize variance in the analysis. The double arcsine transformation has been shown to be preferential to logit transformation or no transformation usage in the calculation of pooled prevalence rates (Barendregt et al., 2013). All analyses were performed using the random-effects model since it allows between-study variation of PVT failure. Forest plots were used to visualize the pooled prevalence of PVT failure, with 95% confidence intervals [CIs]. Where possible, subgroup analyses were performed to examine whether the base rate of PVT failure was related to specific clinical contexts, distinct patient groups, utilized PVT, and the consideration of the presence of potential external gain. To further establish the generalizability of our study findings, the consistency across the included studies was assessed using the Cochran’s Q-test (Higgins et al., 2003). For the Q-test, a *p*-value < 0.10 was considered to indicate statistically significant heterogeneity between studies. Because the number of included studies impacts the Q-test, we additionally evaluated the inconsistency index *I*^2^ (Higgins & Thompson, 2002). An *I*^2^ value over 75% would tentatively be classified as a “high” degree of between-study variance (Higgins et al., 2003). Since *I*^2^ is a relative measure of heterogeneity and its value depends on the precision of included studies, we also calculated Tau squared (*τ*^2^*)*. This measure quantifies the variance of the true effect sizes underlying our data, with larger values suggesting greater between-study variance (Borenstein et al., 2017).

### Study Quality

An adapted version of the Prevalence Critical Appraisal Tool of the Joanna Briggs Institute (Munn et al., 2015) was used to rate the quality of all included studies. Amongst the currently available tools, it addresses the most important items related to the methodological quality when determining prevalence (Migliavaca et al., 2020). Three study quality domains were assessed: selection bias (items 1, 2, and 4), sample size/statistics (items 3 and 5), and attrition bias (item 6; *see* Online Resource 3 for a detailed description).

Doi plot and LFK index are relatively new graphical and quantitative methods that were used for detecting publication bias (Furuya-Kanamori et al., 2018). These analyses were also implemented using MetaXL. Contrary to the scatter plot of precision used in a more standard funnel plot to examine publication bias, the Doi plot uses a quantile plot providing a better visual representation of normality (Wilk & Gnanadesikan, 1968). A symmetric inverted funnel is created with a Z-score closest to zero at its tip if the trials are not affected by publication bias. The LKF index then quantifies the two areas under the Doi plot. The interpretation is based on the a-priori concern about positive or negative publication bias. Since we were concerned about possible positive publication bias, the LFK > 1 was used consistent with positive publication bias. Even in the case of limited included studies, the LKF index has a better sensitivity over the more standard Egger's test (Furuya-Kanamori et al., 2018).

## Results

### Literature Search

**Fig. 1:**
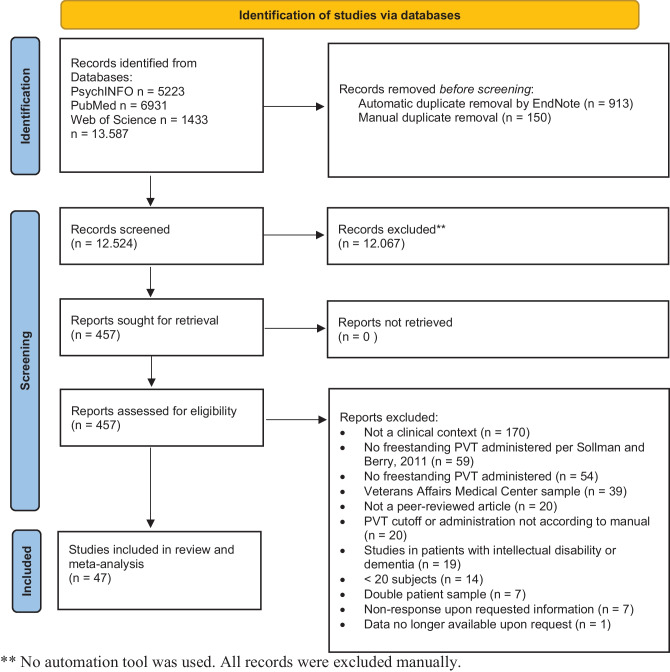
PRISMA flow chart of study selection

Figure 1 gives an overview of the search and selection process. Of the 13,587 identified abstracts, 457 (3.4%) were included for full-text scrutiny. We contacted the first author of 37 studies for additional information, and 30 authors responded. This resulted in 47 observational studies of PVT failure in the clinical context, with a total sample size of *n* = 6,484.

### Characterization of Included Studies

Table 1 reports study characteristics, including clinical context, clinical patient group, and sample size. Most studies were performed in a medical hospital (*k* = 25), with others in an epilepsy clinic (*k* = 7), psychiatric institute (*k* = 6), rehabilitation clinic (*k* = 4), and private practice (*k* = 2). Three studies (6.2%) did not specify clinical context. In 15/47 (31.9%) of the studies, prevalence of PVT failure was reported for heterogeneous patient samples. The majority of the studies (32/47; 68.1%) reported PVT failure rates for one or multiple diagnostic subgroups. The diagnostic (sub)groups constituted of patients with traumatic brain injury (TBI) in most studies (*k* = 10), followed by patients with epilepsy (*k* = 9), patients with psychogenic non-epileptic seizures (PNES; *k* = 5), patients that were seen for attention deficit hyperactivity disorder (ADHD) assessment (*k* = 4), patients with mild cognitive impairment (MCI: *k* = 4), patients with multiple sclerosis (MS; *k* = 2), and patients with Parkinson’s disease (*k* = 2). Severity of TBI was not always specified or was poorly defined. The remaining diagnostic (sub)groups (i.e., sickle cell disease, Huntington’s disease, patients substance-use related disorders (SUD), inpatients with depression, memory complaints) were examined in single studies. In more than half of the included studies (25/47; 53.2%), the language (-proficiency) of the included patient sample was not reported. Potential external gain was not mentioned in 12/47 (25.5%) studies, and the remaining studies varied greatly in how they addressed its presence. Of the remaining 35 studies, only seven (i.e., 20%) specified how external gain was examined (Domen et al., 2020; Eichstaedt et al., 2014; Galioto et al., 2020; Grote et al., 2000; Rhoads et al., 2021a; Williamson et al., 2012; Wodushek & Domen, 2020). In most studies (28/35; 80%), the way the authors examined this variable (e.g., by checking the medical record of patient, querying patients about potential incentives being present during the assessment procedure) was not specified. Moreover, in only 4/35 (11.4%) studies, subjects were excluded when external gain incentives (e.g., workers compensation claim) were present (Dandachi-FitzGerald et al., 2020; Davis & Millis, 2014; Merten et al., 2007; Wodushek & Domen, 2020).

**Table 1:** Summary details for individual studies that reported the prevalence of PVT failure in clinical patients

Study	Type clinical context	Sample type	Sample (*n*)	Mean age (SD)	Mean education (SD)	Country	Language	External incentive	PVT	Administration	PVT failure N [%] ^*^
Cragar et al. (2006)	Epilepsy clinic	Epilepsy	41	36.0 (9.6)	11.9 (3.2)	United States	Not mentioned	'48% on or seeking disability';	TOMM T2 or Retention	Standard	1 (2.4)
									LMT	Computerized	7 (17.1)
		PNES	21	40.8 (10.3)	13.7 (6.1)			'35% on or seeking disability'. Table 1, p. 559	TOMM T2 or Retention	Standard	3 (14.3)
									LMT	Computerized	5 (23.8)
Czornik et al. (2021)	Medical hospital	MCI	28	66.8 (9.9)^a^	12.5 (4.2)^a^	Austria	Not mentioned	'Information about possible secondary gain was not available.' p. 272	WMT IR, DR, or CNS	Computerized German version	2 (7.1)
Dandachi-FitzGerald et al. (2020)	Medical hospital	MCI	41	78.0 (7.2)	[70.7% medium education. Table 1, p. 317]	Netherlands	Dutch	'Involvement in juridical procedures (e.g., litigation)' as exclusion criterion, p. 315	TOMM T2	Dutch version	3/38 (7.9)^g^
		Parkinson's disease	41	63.7 (8.1)	[56.1% medium education. Table 1, p. 317]						1/40 (2.5)^g^
Davis and Millis (2014)	Medical hospital	Heterogeneous neurological	87	42.9 (12.8)^a^	14.0 (2.3)^a^	United States	English, with '19% of the sample reported history of English as a second language', p. 202	'Subjects with potential external incentives were excluded in subgroup analysis.' p. 204	WMT IR, DR, or CNS	Standard	12/58 (20.7)^h^
Deloria et al. (2021)	Private practice	Heterogeneous	181	58.0 (15.7)	13.7 (2.5)	United States	Not mentioned	'7.2% of the sample had indication of involvement in disability or litigation claims.' p. 3	TOMM T2 or Retention	Standard	7/38 (18.4)^i^
Dodrill (2008)	Epilepsy clinic	Epilepsy	65	35.2 (12.9)	12.0 (3.0)	United States	English	Not mentioned	WMT IR, DR, or CNS	Oral version	16 (25.0)
		PNES	32	42.2 (11.6)	12.7 (2.4)						9 (28.0)
Domen et al. (2020)	Medical hospital	MS	84^b^	46.5 (12.9)^b^	14.9 (2.6)^b^	United States	English	'Of note, 16.67% of the analyzed sample endorsed currently applying for disability, and this information was unknown for another 29.76%.' p. 512	MSVT IR, DR, or CNS	Standard	13/108 (12.0)
Donders and Strong (2011)	Rehabilitation clinic	TBI	100	37.5 (13.8)^a^	13.28 (2.3)^a^	United states	English	'(*n* = 28) were involved in disputed financial compensation seeking at the time of the neuropsychological assessment'. p. 176	WMT IR, DR, or CNS	Standard	24 (24.0)
Dorociak et al. (2018)	Medical hospital	Sickle cell disease	54	40.61 (12.3)	13.13 (2.3)	United States	Not mentioned	'None of the subjects was applying for disability or had other known financial incentives related to cognitive status'. p. 85	TOMM T2	Standard	1 (1.9)
Drane et al. (2006)	Epilepsy clinic	Epilepsy	41^a^	36.9 (14.4)	12.6 (2.3)	United States	English	Not mentioned	WMT IR, DR, or CNS	Oral version	3/37 (8.1)
		PNES	43^a^	40.6 (10.2)	12.4 (2.6)						22 (51.2)
Eichstaedt et al. (2014)	Medical hospital	Epilepsy	26	37.8 (11.6)	12.7 (2.6)	United states	English	'Five participants with LTLE reported receiving disability benefits at the time of evaluation, and none failed the WMT.' p. 947	WMT IR, DR, or CNS	Standard	6 (23.1)
Erdodi et al. (2018)	Medical hospital	TBI	104	38.8 (16.7)	13.7 (2.6)	Not mentioned	Not mentioned	'No data were available on litigation status'. p 848	WMT IR, DR, or CNS	Standard	40 (38.5)
Galioto et al. (2020)	Medical hospital	MS	102	47.2 (11.4)	14.4 (2.6)	United States	English	For MS patients only: 27.9% no seeking disability, 38.5% seeking disability, 33.7% already receiving disability, Table 1, p. 1031	VSVT hard items^f^	Standard	15 (14.4)
		Epilepsy	102	47.2 (11.8)	14.3 (2.5)						6 (5.8)
		mTBI	50	42.7 (13.5)	14.3 (2.1)						10 (20.4)
Gorissen et al. (2005)	Mental healthcare institute	Schizophrenia spectrum	64	[Between 18–65. p. 201]	[Less than 6 years of education as exclusion criterion. p. 201]	Netherlands & Spain	Dutch & Spanish	Not mentioned	WMT IR, DR, or CNS	Dutch and Spanish oral versions	46 (72.0)
		Psychiatric (heterogeneous)	63								16 (25.0)
		Neurological (heterogeneous)	20								2 (10.0)
Grote et al. (2000)	Epilepsy clinic	Epilepsy	30	33.4 (10.6)	14.0 (2.6)	Not mentioned	Not mentioned	'They were not seeking compensation at the time of their neuropsychological evaluation. 8 (26.7%) were receiving disability at the time of evaluation because of their seizure disorders.'. p. 711	VSVT hard items	Standard	0
Haber and Fichtenberg (2006)	Rehabilitation clinic	TBI	22	36.4 (13.9)	12.2 (1.4)	United States	Not mentioned	'Subjects were not involved in litigation or workers’ compensation cases.' p. 526	TOMM T2	Standard	0
Haggerty et al. (2007)	Medical hospital	Heterogeneous	300	44.7 (13.0)	13.8 (2.5)	United States	Not mentioned	'Approximately 16% of the sample was involved in litigation and/or seeking compensation for an illness or injury (e.g., workers’ compensation, disability) at the time of their evaluations.' p. 921	VSVT hard items	Standard	24 (8.0)
Harrison and Armstrong (2020)	Mental healthcare institute	ADHD	245	20.4 (1.8)	[All participants were students. '57.1% in their first or second year.' p. 316]	Canada	Not mentioned	Not mentioned	MSVT IR, DR, or CNS	Standard	49 (20.0)
Harrison et al. (2021)	Mental healthcare institute	ADHD	2463	21.8 (5.9)	['All students were high school graduates or equivalent, with their college or university program in progress.' p. 2]	Canada	Not mentioned	'All were seeking a diagnosis to allow access to disability supports and services'. p. 3	MSVT IR, DR, or CNS	Standard	57/648 (8.8)
									WMT IR, DR, or CNS	Standard	206/1810 (11.4)
Hoskins et al. (2010)	Epilepsy clinic	Epilepsy	31	38.5 (12.0)	12.7 (2.7)	United States	English	Not mentioned	WMT IR, DR, or CNS	Oral version (*n* = 30); computerized version (*n* = 31)	7 (22.6)^j^
		PNES	30								11 (36.7)^j^
Jennette et al. (2021)	Medical hospital	Heterogeneous	128	45.7 (16.4)	14.0 (2.6)	United States	English,14% bilingual English. Table 1, p. 4	14% compensation seeking. Table 1, p. 4	MSVT IR, DR, or CNS	Standard	32 (25.0)
Keary et al. (2013)	Epilepsy clinic	Epilepsy	404	38.5 (12.1)	13.3 (2.1)	United States	Not mentioned	'None of the patients in this clinically referred sample were known to be involved in litigation regarding their medical status or seeking financial compensation at the time of their neuropsychological evaluations.' p. 315	VSVT hard items^f^	Standard	22 (5.4)
Krishnan and Donders (2011)	Rehabilitation clinic	TBI	115	40.71 (13.3)	12.86 (2.2)	United States	Not mentioned	32% seeking financial compensation. Table 1, p. 179	TOMM T2 or Retention	Standard	3/39 (8)
									WMT IR, DR, or CNS	Standard	25/81 (31)
Leppma et al. (2018)	Mental health care institute	ADHD	350	22.6	[22.9% Graduate Students. p. 213]	United States	Not mentioned	Not mentioned	NV-MSVT IR, DR, or CNS	Standard	68 (21.1)
Locke et al. (2008)	Medical hospital	Acquired brain injury	87	36.3 (12.2)	13.4 (2.5)	United States	Not mentioned	'76% of the sample was on disability at the time of the evaluation.' p. 275	TOMM T2	Standard	19 (21.8)
Loring et al. (2007)	Medical hospital	Neurological (heterogeneous)	27	47.4 (13.2)	13.9 (2.4)	United States	Not mentioned	'No known external financial incentive'. p. 524	VSVT hard items	Standard	2 (7.0)
		Memory complaints	163	51.8 (13.0)	13.8 (2.6)						16 (10.0)
		TBI	49	36.7 (10.8)	12.7 (1.9)						6 (12.0)
Loring et al. (2005)	Medical hospital	Epilepsy	120	34.5 (11.1)	12.6 (2.2)	United States	Not mentioned	'Not actively screened for compensation status.' (p. 611)	VSVT hard items^f^	Standard	14 (11.7)
Marshall et al. (2016)	Mental health care institute	ADHD	428	26.4 (7.8)	14.4 (1.9)	United States	Not mentioned	Not mentioned	WMT IR, DR, or CNS	Standard	53/174 (30.4)^k^
Martins and Martins (2010)	Not specified	MCI	21	71.2 (2.0)	[71.4% had less then 6 years of education. Table 1, p. 178]	Portugal	Portuguese	'None of these patients had any identifiable secondary gain. All patients were retired and without ongoing legal processes.' p. 178	WMT IR, DR, or CNS	Portuguese computerized version	14 (67.0)
Merten et al. (2007)	Medical hospital	Heterogeneous	48	56.4 (13.1)^a^	[Minimum of 8 years of formal schooling as inclusion criterion. p. 309]^a^	Germany	German	'Involvement in litigation' as exclusion criterion.’ p. 309	TOMM T2 or Retention	German version	1/24 (4.2)^c^
									WMT IR, DR, or CNS	Oral, German version	2/24 (8.3)
Meyers et al. (2014)	Not specified	Heterogeneous	255	34.5 (12.1)	12.5 (1.8)	United states	Not mentioned	'76 [subjects] were in litigation' p. 225	WMT, IR, DR, or CNS	Standard	98 (38.4)
Moore and Donders (2004)	Rehabilitation clinic	TBI	132	35.8 (14.2)	12.3 (2.6)	United States	Not mentioned	'Those seeking financial compensation (*n* = 26) were not excluded.' p. 977	TOMM T2	Standard	11 (8.3)
Neale et al. (2022)	Medical hospital	Heterogeneous	147	46.4 (14.5)	13.2 (2.2)	United States	Not mentioned	20/147 (13.6%) were seeking compensation. Table 2, p. 5	MSVT IR, DR, or CNS	Standard	30/145 (20.7)^l^
Rees et al. (2001)	Medical hospital	Inpatients with depression	26	40.4 (11.2)	4.9 (2.8)	Canada	English	Not mentioned	TOMM T2 or Retention	Standard	0
		TBI	24	40.4 (14)	13.6 (2.7)						0
Resch et al. (2021)	Not specified	Heterogeneous	88	31.7 (10.2)	15.4 (2.3)	United States	English	Not mentioned	TOMM T2	Standard	10/33 (30.0)
Rhoads et al. (2021a)	Medical hospital	Heterogeneous	132	45.1 (16.3)	14.0 (2.6)	United States	English	'Finally, 15% of patients (*n* = 20) reported being concurrently compensation-seeking (e.g., disability) at the time of their clinical evaluation' p. 135–136	MSVT IR, DR, or CNS	Standard	28 (21.2)^m^
Rhoads et al. (2021b)	Medical hospital	Heterogeneous	112	60.6 (15.9)	8.1 (4.5)	United States	Spanish	*n* = 20 (17.9%) compensation seeking. Table 3, p. 272	TOMM T2	Spanish version	20/86 (23.3)
Sabelli et al. (2021)	Private practice	mTBI	326	39.5 (11.8)	12.1 (2.6)	Canada	Not mentioned	Not mentioned	WMT IR, DR, or CNS	Standard	104 (31.9)
Schroeder et al. (2019)	Medical hospital	Heterogeneous	162	46.4 (13.2)	13.6 (2.3)	United States	Not mentioned	'Roughly 65% of the sample had known or suspected secondary gain associated with the evaluation. The secondary gain was most commonly related to a pursuit of: disability, civil litigation, or workers compensation.', p. 468	TOMM T2 or Retention	Standard	25 (15.0)
Sharland et al. (2018)	Medical hospital	Heterogeneous	615	43.4 (12.8)	12.6 (2.6)	United States	English	'Additionally, while the sample was clinical in nature, it is possible a proportion of participants were also involved in litigation, applying for disability, or on workers compensation'. p.105	TOMM T2 or Retention	Standard	49 (7.9)^n^
Sieck et al. (2013)	Medical hospital	Huntington disease	36	46.1 (12.6)	13.6 (2.2)	United States	Not mentioned	Not mentioned	TOMM T2	Standard	3 (8.2)
Silverberg et al. (2017)	Medical hospital	mTBI	80	40.8 (12.0)	[*n* = 37 (46.3%) with postsecondary degree. Table 1, p. 2143]	United States	English	*n* = 71 (86.4%) receiving or seeking injury compensation. Table 1, p. 2143	MSVT IR, DR, or CNS	Standard	20 (25.0)
Teichner and Wagner (2004)	Medical hospital	Cognitive impairment, not demented	36	70.6 (8.1)	14.2 (3.2)	United States	Not mentioned	Not mentioned	TOMM T2 or Retention	Standard	3 (8.3)
		Cognitively intact	21	65.6 (8.6)	14.2 (3.6)						0
Vilar-López et al. (2021)	Mental healthcare institute	Substance-use related disorders (SUD)	77	43.2 (8.2)^d^	[patients with primary schooling (*n* = 35) constituted the largest category. Table 1, p. 257]	Spain	Spanish	' The second group, made up of SUD patients with compensation seeking (*n* = 36), completed a neuropsychological evaluation in order to apply for economic compensation due to their disability (according to their disability level, participants could obtain a monthly payment for life or a payment reviewable at 4 years).' p. 256–257	TOMM T2 and Retention	Spanish version	1 (1.3)
Walter et al. (2014)	Medical hospital	MCI	31	66.0 (8.0)	14.7 (2.1)	United states	Not mentioned	'No participant was involved in litigation at the time of the evaluation or had a substantial external incentive to perform poorly.' p .1200	TOMM T2	Standard	3 (9.7)
Wodushek and Domen, (2020)	Medical hospital	Parkinson's disease	55	65.2 (8.9)	14.9 (2.8)	United States	English	'Cases were excluded from analysis if the patient reported being involved in litigation, or if there was an obvious external or secondary gain issue, such as a disability application (*n* = 1).' p. 11	MSVT IR, DR, or CNS	Standard	5/51 (9.8)
Williamson et al. (2012)	Epilepsy clinic	PNES	90	39.0 (8.3)^e^	13.2 (2.0)^e^	United States	English	'The presence of financial incentives was determined on the basis of patient report. Patients were classified as having financial incentives if they were currently receiving or applying for disability benefits or other forms of financial compensation (e.g., worker’s compensation)'. p. 591	WMT IR, DR, or CNS	Oral version	32 (35.5)

*ADHD* attention deficit hyperactivity disorder; *CNS* consistency score; *DR* delayed recognition; *IR* immediate recognition; *LMT* letter memory test; *MCI* mild cognitive impairment; *MS* multiple sclerosis; *(m)TBI* (mild) traumatic brain injury; *(nv)MSVT* (non-verbal) medical symptom validity test; *PNES* psychogenic non-epileptic seizures; *T2* trial 2; *TOMM* test of memory malingering; *VSVT* Victoria symptom validity test; *WMT* word memory test

^*^ In case not all subjects in the patient sample received a given PVT, the proportion is shown in this column (…/…)

^a^ The authors provided demographics for the total patient sample, not for the (sub)samples for which PVT failure rates were reported

^b^ The authors only provided demographics for the final sample, after excluding cases with missing data. The initial total sample that was provided with a PVT was larger than the sample size detailed in the article

^c^ Results of the "no clinically obvious cognitive impairment" subgroup (*n* = 24)

^d^ Demographics for the total sample (*n* = 77) were not mentioned. Therefore, we choose to display the demographics of the non-compensation subgroup (*n* = 41)

^e^ Demographics for the total sample (*n* = 90) were not mentioned. Therefore, we choose to display the demographics of the WMT fail subgroup (*n* = 32)

^f^ VSVT hard items cutoff per Grote et al., 2000 in epilepsy (sub)sample

^g^ B. Dandachi-Fitzgerald (personal communication, February 4, 2022)

^h^ J. Davis (personal communication, February 26, 2021)

^i^ A. Kivisto (personal communication, February 9, 2022)

^j^ D. Drane (personal communication, June 30, 2021)

^k^ P. Marshall (personal communication, February 26, 2021)

^l^ A. Neale (personal communication, February 5, 2022)

^m^ T. Rhoads (personal communication, December 4, 2021)

^n^ M. Sharland (personal communication, February 4, 2022)

The TOMM was the most frequently administered PVT (*k* = 18), followed by the WMT (*k* = 17), the MSVT (*k* = 9), the VSVT (*k* = 6), or the LMT (*k* = 1). Only 4/47 (8.5%) studies employed two PVTs (none used > 2 PVTs that fulfilled the inclusion/and exclusion criteria). The other 43/47 (91.5%) studies used one PVT. In two of the four studies reporting two PVTs, the same PVTs were not administered to all participants. Harrison et al. (2021) administered the MSVT to 648 patients and the WMT to 1810 patients, and Krishnan and Donders (2011) administered the TOMM to 39 patients and the WMT to 81 patients. Inclusion in these studies was – amongst others – based upon failing one PVT. Furthermore, these studies did not report the number of subjects that were provided with both PVTs. Therefore, it is unclear to what extend the reported PVT failure rates in these studies are influenced by potential dependence. In the two other studies reporting two PVTs (i.e., Cragar et al., 2006; Merten et al., 2007), all patients were administered both PVTs. The total number of subjects in these two studies that reported two likely dependent effect-sizes was *n* = 76. This is 1.2% of the total of *n* = 6487 patients from all 47 studies. We therefore argue that the reported effect sizes from the 47 included studies are (largely) independent.

### Methodological Quality Assessment

A summary of the methodological quality of the included studies for determining prevalence is provided in Online Resource 4. No study was rated as having high quality; all had limitations in at least one of the three prespecified domains (selection bias, attrition bias, and sample size/statistical analyses). Most studies had a study sample that addressed the target population (*k* = 41, 87.2%), whereas only a minority described relevant assessment and patient characteristics (*n* = 15, 31.9%). The majority of included studies failed to clearly state how patients were recruited (*n* = 27, 57.4%). Eleven studies (23.4%) had an inadequate response rate. The majority of the studies used appropriate statistical analyses (*n* = 41, 87.2%), but also had inappropriate sample sizes (*n* = 39, 83.0%).

The shape of the Doi plot showed slight asymmetry (*see* Online Resource 5), and the results of the LFK index (1.09) revealed minor asymmetry indicative of potential positive publication bias.

### Base Rate of PVT Failure in Clinical Patients

**Fig. 2:**
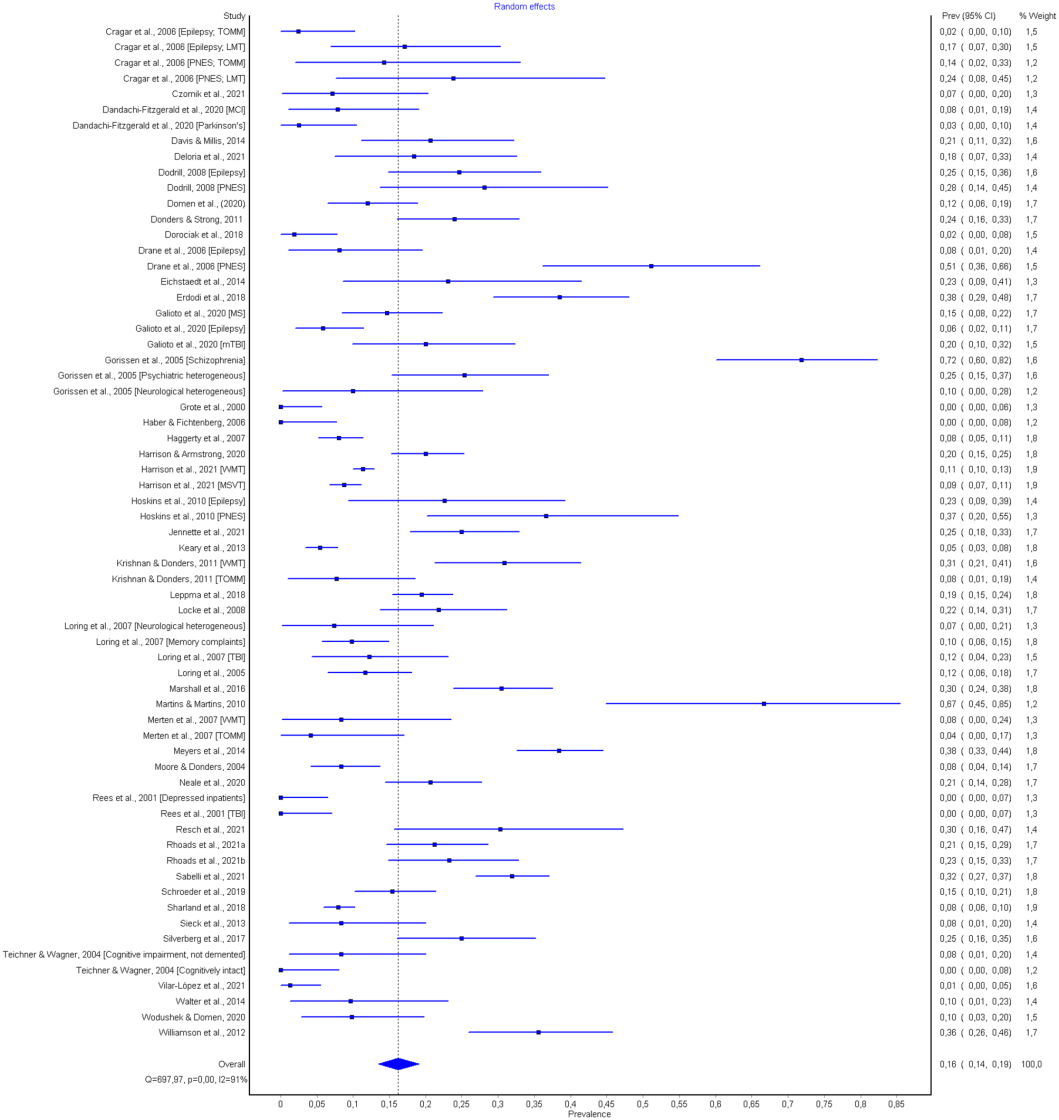
Forest plot of the 47 included studies estimating the pooled prevalence of PVT failure in the clinical setting CI = confidence interval. Note: Weights are from random effects analysis

The pooled prevalence of PVT failure of all (*n* = 47) included studies was 16%, 95% CI [14, 19]. Significant between-study heterogeneity and high between-study variability existed (Cochran's Q = 697.97, *p* < 0.001; *I*^2^ = 91%; τ^2^ = 0.08) as revealed by the large 95% CIs (see Fig. 2). The high *I*^2^ statistic indicates that the variation in reported PVT failure is likely a result of true heterogeneity rather than chance.

### Subgroup Analyses based upon Clinically Relevant Characteristics

To facilitate the interpretation of PVT failure in clinical practice, subgroup analyses were performed for clinically relevant characteristics associated with performance validity (Table 2). It is important to emphasize that some of these findings are based upon relatively small numbers of studies (i.e., *k* = 2 or 4), potentially impacting the stability if the reported estimates.

**Table 2:** Pooled prevalence of PVT failure in clinical patients, stratified by false-positive scrutinization, clinical context, external gain incentives, clinical diagnosis, and PVT

Clinical characteristics	*n/k*	Pooled PVT failure rate (%)	95% CI	*I*^2^(%)	τ^2^
Overall	6,484/47	16	14–19	91	0.08
False-positive scrutinization					
Probable risk of false-positive PVT failure classification	85/2	70	60–80	0	0.00
Probable no risk of false-positive PVT failure classification	6,399/46	15	13–18	89	0.07
Clinical setting*					
Private practice	364/2	27	15–40	66	0.03
Epilepsy clinic	824/7	19	10–29	91	0.17
Mental healthcare institute	1,577/6	15	10–24	92	0.04
Medical hospital	3,057/25	12	10–15	81	0.05
Rehabilitation clinic	293/4	13	4–25	88	0.10
Subjects with potential external gain incentives excluded?*					
Yes	211/4	10	5–15	45	0.02
No	6,188/42	16	13–19	90	0.07
Clinical diagnosis*					
PNES	216/5	33	24–43	53	0.03
ADHD	1,417/4	17	11–23	94	0.03
(m)TBI	926/10	17	10–25	89	0.09
MS	210/2	13	9–18	0	0.00
Epilepsy	856/9	11	6–16	79	0.05
MCI	97/3	9	4–16	0	0.00
Parkinson’s disease	91/2	6	1–15	45	0.02
PVT*					
WMT	1,482/13	25	19–32	93	0.10
(nv)MSVT	1,891/9	18	13–23	85	0.03
TOMM	1,759/18	9	6–12	80	0.05
VSVT	1,347/6	9	7–2	64	0.01

*ADHD* attention deficit hyperactivity disorder; *CI* confidence interval; *MCI* mild cognitive impairment; *MS* multiple sclerosis; *(m)TBI* (mild) traumatic brain injury; *(nv)MSVT* (non-verbal) medical symptom validity test; *PNES* psychogenic non-epileptic seizures; *PVT* performance validity test; *TOMM* test of memory malingering; *VSVT* Victoria symptom validity test; *WMT* word memory test. * after exclusion of the subsamples of subjects with a probable risk of false-positive PVT failure classification (i.e., *k* = 46)

#### False-Positive Scrutinization

Although we excluded studies that examined PVT failure rates in patients with dementia or intellectual disability *a priori*, the included studies might still comprise patient samples with other conditions or combinations of characteristics that make them highly susceptible to false-positive PVT failure classification. Therefore, and in line with clinical guidelines (Sweet et al., 2021), we first examined included studies for the risk of unacceptably low specificity rates when applying standard PVTs cutoffs, and two studies were identified. First, PVT performance in the subsample of severely ill schizophrenia spectrum and mostly inpatients from Gorissen et al. (2005) was significantly correlated with negative symptoms and general psychopathology. Second, the MCI subjects from Martins and Martins (2010) were of advanced age, Spanish speaking, and had the lowest formal schooling of all included studies (i.e., 71.4% had less than 6 years of formal education). These cultural/language factors in combination with low formal schooling are associated with unacceptably low specificity rates when applying standard PVT cutoffs (Robles et al., 2015; Ruiz et al., 2020). Exclusion of the subsample of patients with schizophrenia in the Gorissen et al. (2005 study) and of the Martins and Martins (2010) study led to a pooled prevalence of PVT failure of 15% (95% CI [13, 18]; Cochran's Q = 573.73, *p* < 0.01; *I*^2^ = 89%; τ^2^ = 0.07). However, after exclusion of these patient samples, between-study heterogeneity and between-study variability were still high as indicated by a significant Cochran's Q statistic and high and *I*^2^ statistic. Further subgroup analyses were performed in the remaining studies (*k* = 46; see Table 1).

#### Clinical Context

The pooled prevalence of PVT failure was the highest in the context of a private practice (27%, 95% CI [15, 40]; Cochran's Q = 2.98, *p* = 0.08, *I*^2^ = 66%; τ^2^ = 0.03), followed by the epilepsy clinic (19%, 95% CI [10, 29]; Cochran's Q = 128.07, *p* < 0.001, *I*^2^ = 91%; τ^2^ = 0.17), the psychiatric institute (15%, 95% CI [10, 21]; Cochran's Q = 92.30, *p* < 0.001, *I*^2^ = 92%; τ^2^ = 0.04), the medical hospital (12%, 95% CI [10, 15]; Cochran's Q = 160.51, *p* < 0.001, *I*^2^ = 81%; τ^2^ = 0.05) and the rehabilitation clinic (13%, 95% CI [4, 25]; Cochran's Q = 31.07, *p* < 0.001, *I*^2^ = 88%; τ^2^ = 0.10). As can be seen, heterogeneity of pooled PVT failure rates was significant and between-study variability was moderately-high to high for all types of clinical context.

#### Clinical Diagnoses

The pooled prevalence of PVT failure was the highest for patients with PNES (33%, 95% CI [ 24, 43]; Cochran's Q = 10.65, *p* = 0.06; *I*^2^ = 53%; τ^2^ = 0.03), followed by subjects seen for ADHD assessment (17%, 95% CI [11, 23]; Cochran's Q = 68.80, *p* < 0.01; *I*^2^ = 94%; τ^2^ = 0.03), (m)TBI (17%, 95% CI [10, 25]; Cochran's Q = 89.57, *p* < 0.01; *I*^2^ = 89%; τ^2^ = 0.09), MS (13%, 95% CI [9, 18]; Cochran's Q = 0.32, *p* = 0.57; *I*^2^ = 0%; τ^2^ = 0.00), epilepsy (11%, 95% CI [6, 16]; Cochran's Q = 42.21, *p* < 0.001; *I*^2^ = 79%; τ^2^ = 0.05), MCI (9%, 95% CI [4, 16]; Cochran's Q = 0.11, *p* = 0.95; *I*^2^ = 0%; τ^2^ = 0.00), and Parkinson's disease (6%, 95% CI [1, 15]; Cochran's Q = 1.81, *p* = 0.18; *I*^2^ = 45%; τ^2^ = 0.02). Based upon Cochran's Q, heterogeneity of pooled PVT failure rates was significant in patients with PNES, (m)TBI, epilepsy, Parkinson's disease, and subjects seen for ADHD assessment. Non-significant heterogeneity in pooled PVT failure rates was found in patients with Parkinson's disease, MCI, and MS. Based upon the *I*^2^ statistic, variability of base rate estimates of PVT failure was low in patients with MCI and MS, and (moderately) high for the other diagnostic patient groups. This suggests that for studies in patients with MCI and MS, the pooled PVT failure rates are more homogeneous. However, since these calculations are based upon small numbers of studies, these findings should be interpreted with caution (von Hippel, 2015).

#### External Gain Incentives

In the four studies where patients with potential external gain incentives were excluded from analysis, the pooled prevalence of PVT failure was as low as 10% (95% CI [5, 15]; Cochran's Q = 9.17, *p* = 0.10; *I*^2^ = 45%; τ^2^ = 0.02). For the 42 remaining studies that did not report to have actively excluded clinical patients with potential external gain incentives before reporting PVT failure, however, the pooled prevalence of reported PVT failure was 16% (95% CI [13, 19]; Cochran's Q = 560.93, *p* < 0.001; *I*^2^ = 90%; τ^2^ = 0.07). Although Cochran's Q statistic indicated that heterogeneity of pooled PVT failure rates in both groups was high, inconsistency was lower in the studies where patients with external gain were excluded from analysis.

#### PVT

The pooled prevalence of PVT failure was the highest for patients examined with the WMT (25%, 95% CI [19, 32]; Cochran's Q = 253.52, *p* < 0.001; *I*^2^ = 93%; τ^2^ = 0.10), followed by the (nv)MSVT (18%, 95% CI [13, 23]; Cochran's Q = 55.04, *p* < 0.001, *I*^2^ = 85; τ^2^ = 0.03), the TOMM (9%, 95% CI [6, 12]; Cochran's Q = 103.35, *p* < 0.001, *I*^2^ = 80; τ^2^ = 0.05), and the hard items of the VSVT (9%, 95% CI [7, 12]; Cochran's Q = 24.97, *p* < 0.001, *I*^2^ = 64; τ^2^ = 0.01). Heterogeneity of pooled PVT failure rates was significant across studies examining the same PVT, whereas the between-study variability was moderately-high for studies using the VSVT and high in studies using other PVTs.

## Discussion

This systematic review and meta-analysis examined the prevalence of PVT failure in the context of routine clinical care. Based on extracted data from all 47 studies involving 6,484 patients seen for clinical assessment, the pooled prevalence of PVT failure was 16%, 95% CI [14, 19]. Excluding two studies that likely represented patients where standard PVT cutoff application would probably lead to false positive classification, resulted in a pooled PVT failure of 15%, 95% CI [13, 18]. This number corresponds with the median estimated base rate of invalid performance in clinical settings reported in a recent survey amongst 178 adult-focused neuropsychologists (Martin & Schroeder, 2020). Our empirical findings confirm PVT failure in a sizeable minority of patients seen for clinical neuropsychological assessment.

Another key finding is that reported PVT failure rates vary significantly amongst the included studies (i.e., 0–52.2%). This variability is likely due to (1) sample characteristics, such as clinical setting, clinical diagnosis, and potential external incentives, and (2) the sensitivity and specificity of the PVT used. Pooled PVT failure was found to be highest (i.e., 27%, 95% CI [15, 40]) in patients seen in private practice. The pooled PVT failure rates for the other settings (i.e., epilepsy clinic, psychiatric institute, medical hospital, and rehabilitation clinic) varied between 13–19%. The Sabelli et al. (2021) study had the largest private practice sample (*N* = 326), consisting of relatively young mTBI patients referred for neuropsychological evaluation. Since only 2/47 of the included studies were conducted in the private practice setting, the Sabelli et al. (2021) study with a PVT failure rate of 31.9%, was a major contributor to the higher pooled PVT failure rate in a private practice setting. Of interest, potential external incentives were not mentioned in that study. Therefore, potential external gain incentives may have been present and impacted the relatively high level of PVT failure rather than assessment context per se. Unsurprisingly, but now clearly objectified, studies that excluded patients with potential external gain incentives had a significantly lower pooled PVT failure rate compared to studies where these subjects (potentially) remained in the analysis (i.e., 10%, 95% CI [ 5, 15] versus 16%, 95% CI [13, 19] respectively). However, although it is known that the presence of external incentive links directly to PVT failure in clinical evaluations (e.g., Schroeder et al., 2021b), little over a quarter of the included studies failed to mention the presence of external gain incentives. Moreover, even when external gain incentives were known to be present, only a minority of studies excluded these subjects from further analyses. Pooled PVT failure rates were highest for patients diagnosed with PNES (i.e., 33%, 95% CI [24, 43]), patients seen for ADHD assessment (i.e., 17%, 95% CI [11, 23]), and (m)TBI (i.e., 17%, 95% CI [10, 25) with pooled PVT failure rates ranging between 6–13% for the other diagnostic groups (i.e., MS, epilepsy, MCI, and Parkinson's disease). These findings contrast with McWhirter et al. (2020), who reported PVT failure in subjects with functional neurological disorders (such as PNES) are no higher compared to MCI or epilepsy. Likely, our strict inclusion and exclusion criteria, inclusion of only well-validated stand-alone PVTs, and meta-analysis application lead to a more precise estimate of PVT failure across diagnostic groups. Our findings also indicate that pooled PVT failure rates for MCI, MS, and Parkinson's disease diagnostic groups are more homogeneous than those of PNES, (m)TBI, and patients seen for ADHD assessment. The higher levels of heterogeneity in these latter groups could indicate that other factors that likely impact PVT failure were present, such as external gain incentives, variation in diagnostic criteria, and bias in patient selection. Finally, pooled failure rates varied across the utilized PVTs in line with their respective sensitivity/specificity ratios in correctly identifying invalid performance. The WMT is known for its relatively high sensitivity (Sollman & Berry, 2011), which likely resulted in the highest pooled failure rate amongst the examined stand-alone PVTs. The lowest pooled failure rate for the TOMM is probably related to its high specificity (Martin et al., 2020).

Our findings indicate that in addition to PVT psychometric properties (i.e., sensitivity and specificity), the clinical setting, the presence of external gain incentives, and the clinical diagnosis impact pooled PVT failure rates. The clinician should therefore consider these factors when interpreting PVT results. Consider, for example, a well-researched stand-alone PVT with a sensitivity of 0.69 and specificity of 0.90, administered to two different clinical patients. The first patient is diagnosed with epilepsy and wants to get approved to return to work (i.e., no external gain incentives for invalid performance). If the mentioned PVT were failed in the context of this patient without external gain incentives (base rate PVT failure of 10%, *see* Table 2), the likelihood that PVT failure was indeed a true positive (i.e., positive predictive value, PPV) would be 43%. The second patient is also diagnosed with epilepsy but has a pending disability application because the patient does not believe he/she is able to return to work (i.e., potential external gain incentive for invalid performance). If the same PVT were failed in the context of this patient with potential external gain incentive (base rate PVT failure of 16%, *see* Table 2), PPV would be 57%.

Of importance, although a PPV increase of 0.43 to 0.57 is substantial, the latter is still not sufficient to determine performance validity. Therefore, in line with general consensus multiple, independent, validity tests should be employed (Sherman et al., 2020; Sweet et al., 2021). By chaining the positive likelihood-ratios (LRs) of multiple failed PVTs, the diagnostic probability of invalid performance (or PPV) is increased and the diagnostic error is decreased (for an explanation of how to chain likelihood ratios *see* Larrabee, 2014; Larrabee, 2022). Note that while considerable weight should be placed on the psychometric evaluation of performance validity, the clinician should also include other test and extra-test information (e.g., degree of PVT failure, (in)consistency of the clinical presentation) to draw conclusions about the validity of an individual patient's neuropsychological assessment (Dandachi-FitzGerald & Martin, 2022; Larrabee, 2022; Sherman et al., 2020).

Strengths of the present study are its strict inclusion/ exclusion criteria ensuring accurate PVT results. Unfortunately, none of the studies fulfilled all components of the three pre-defined quality criteria selection bias, attrition bias, and adequate sample size/statistics for determining prevalence. Although we excluded studies with < 20 subjects, most of the remaining studies still relatively small sample sizes, increasing the likelihood of sampling bias and heterogeneity. Moreover, only 20/47 studies reported appropriate recruitment method (e.g., consecutive referrals of a good census) necessary for determining the base rate of PVT failure. Additionally, diagnostic criteria varied across studies, limiting the generalizability of their calculated PVT failure base rates. Also, the way potential external gain incentives were examined and defined varied significantly. Surprisingly, in just over one quarter of included studies, potential external gain incentives were not mentioned at all, and potential external gain incentives may have been present. Finally, although language (-proficiency) and cultural factors relate to PVT failure (Robles et al., 2015; Ruiz et al., 2020), these factors were not mentioned in more than half of the included studies in our meta-analysis.

Additional empirical research is necessary to advance knowledge of performance validity test failure in clinical populations. An important first step in future research should be to provide comprehensive details regarding study design, such as recruitment procedure, clinical setting, and demographic/descriptive information (e.g., cultural factors, age, language and language proficiency, level of education). A second improvement would be to form comparable and homogeneous patient samples by specifying diagnostic criteria and providing a detailed specification of how external gain incentives were examined (e.g., querying the patient for potential external gain incentives, such as pending litigation or disability procedures; Schroeder et al., 2021a). Since administration of multiple PVTs is recommended (Sweet et al., 2021), future studies and specifically meta-analyses should consider using advanced statistical techniques (e.g., three-level meta-analyses) in handling non-independent effect sizes (Cheung, 2019).

In conclusion, the current meta-analysis demonstrates that PVT failure occurs in a substantial minority of patients seen for routine clinical care. Type of clinical context, patient characteristics, presence of external gain incentives, and psychometric properties of the utilized PVT are found to impact the rate of PVT failure. Our findings can be used for calculating clinically applied statistics (i.e., PPV/NPV, and LRs) in everyday practice to increase the diagnostic accuracy of performance validity determination. Future studies using detailed recruitment procedures and sample characteristics, such as external gain incentives and language (proficiency), are needed to further improve and refine knowledge about the base rates of PVT failure in clinical assessments.

### Supplementary Information

Below is the link to the electronic supplementary material.

Supplementary file1 (DOCX 13 KB)

Supplementary file2 (DOCX 16 KB)

Supplementary file3 (DOCX 16 KB)

Supplementary file4 (DOCX 20 KB)

Supplementary file5 (DOCX 19 KB)

## Data Availability

Data are available upon reasonable request from the first author.
