# Supplementary material for: Performance Validity Test Failure in the Clinical Population: A Systematic Review and Meta-Analysis of Prevalence Rates
Source: Neuropsychol Rev. 2023 Mar 6;34(1):299–319. doi: 10.1007/s11065-023-09582-7 (PMC10920461; doi:10.1007/s11065-023-09582-7)
Supplement: Supplementary file 1 — Supplementary file1 (DOCX 13 KB) [file 11065_2023_9582_MOESM1_ESM.docx]

**Online Resource 1: Detailed search strategies**

**Search Strategy in PubMed**

**(((((((((((((malingering[MeSH Terms] OR "symptom validity" OR svt OR effort OR underperformance OR "invalid performance" OR "performance validity" OR pvt))) AND (("neuropsychological tests"[MeSH Terms] OR "cognition disorders"[MeSH Terms] OR neuropsych*))) AND ((dutch[lang] OR german[lang] OR english[lang]))) AND humans[MeSH Terms]) AND adult[MeSH Terms]) NOT child[MeSH Terms]))))))**

**Search strategy in PsychINFO**

S1: malingering OR "symptom validity" OR svt OR effort OR underperformance OR "invalid performance" OR "performance validity" OR pvt

S2: "neuropsychological test*" OR cognition OR "neuropsychological assessment"

**Limiters** - Published Date: -20211131; Publication Type: Peer Reviewed Journal; Language: Dutch, English, German; Age Groups: Adulthood (18 yrs & older), Young Adulthood (18-29 yrs), Thirties (30-39 yrs), Middle Age (40-64 yrs), Aged (65 yrs & older), Very Old (85 yrs & older); Population Group: Human

**Search strategy in Web of Science**

((TS=(malingering OR "symptom validity" OR svt OR underperformance OR "invalid performance" OR "performance validity" OR pvt )) AND TS=("neuropsychology*" OR "cognit*" )) NOT TS=("animal*")

*[NB: Search term “effort” was dropped here due to too many hits.]*
