# Supplementary material for: Performance Validity Test Failure in the Clinical Population: A Systematic Review and Meta-Analysis of Prevalence Rates
Source: Neuropsychol Rev. 2023 Mar 6;34(1):299–319. doi: 10.1007/s11065-023-09582-7 (PMC10920461; doi:10.1007/s11065-023-09582-7)
Supplement: Supplementary file 2 — Supplementary file2 (DOCX 16 KB) [file 11065_2023_9582_MOESM2_ESM.docx]

**Online Resource 2: Data Collection Form**

Data Collection Form

‘Prevalence Invalid Performance Clinical Context’

**Version and date**: 4, 2020

| Study ID *(surname of first author and year first full report of study was published e.g. Smith 2001)* |  |
| --- | --- |
| Abstract ID *(from endnote library)* |  |

# Study Characteristics // Methods

| **Type of study** | case-control  cross-sectional retrospective cohort |
| --- | --- |
| **Participants** (N, mean age/SD, level of education, language, country) |  |
| **Population description *(from which study participants are drawn)*** |  |
| **Diagnoses (specify when heterogeneous)** |  |
| **Clinical setting** **(evaluation context)** | Medical hospital  Mental Health Care Inst.    Specialized clinic (eg. epilepsy)  Private practice  Other |
| **Specify setting ‘other’** |  |
| **Inclusion criteria** |  |
| **Exclusion criteria** |  |
| **Confounding variables low IQ and/or severe cognitive impairment mentioned?** | YES  NO |

| **External gain known?** | YES NO |
| --- | --- |
| **How is external gain known?** | Assumed based on context of assessment (i.e., not in litigation etc.)  Other |
| **Specify external gain ‘other’.** |  |

| **PVT(s) *(specify when > 1 PVT used)*** |  |
| --- | --- |
| **Utilized cut-off's** |  |
| **Administered in line with manual?** | YES NO **(= possible exclusion!)**  Not stated |
| **% PVT failure *(specify for every diagnostic and external gain group when possible)*** |  |
| **PVTs mean/SD/score-range** |  |

| **Correspondence for further study information (fill out when necessary)** |  |
| --- | --- |
