# Supplementary material for: Performance Validity Test Failure in the Clinical Population: A Systematic Review and Meta-Analysis of Prevalence Rates
Source: Neuropsychol Rev. 2023 Mar 6;34(1):299–319. doi: 10.1007/s11065-023-09582-7 (PMC10920461; doi:10.1007/s11065-023-09582-7)
Supplement: Supplementary file 3 — Supplementary file3 (DOCX 16 KB) [file 11065_2023_9582_MOESM3_ESM.docx]

**Online Resource 3**

**Adapted version of the Joanna Briggs Institute (JBI) Critical Appraisal Checklist for studies Reporting Prevalence Data to rate Study Quality**

1. **Was the sample frame appropriate to address the target population?**

The in- and exclusion criteria ensured that the sample frame was appropriate to address the target population (i.e., patients seen for routine care in a clinical context). In case additional diagnostic (sub) groups were examined, the following diagnostic criteria were used to examine adequate diagnostic (sub)group allocation.

For MCI, we used the diagnostic criteria from Petersen, R. C., Smith, G. E., Waring, S. C., Ivnik, R. J., Tangalos, E. G., & Kokmen, E. (1999). Mild cognitive impairment: Clinical characterization and outcome. *Archives of Neurology, 56*, 303–308.

For (m)TBI, we used presence of one or more of the following DSM-V criteria: (1) loss of consciousness, (2) posttraumatic amnesia (PTA), (3) disorientation and confusion, and (4) neurological signs (e.g., visual field cuts, or new onset of seizures) (American Psychiatric Association. (2013). Neurocognitive disorders. In *Diagnostic and statistical manual of mental disorders* (5th ed.)

For epilepsy diagnosis, seizures needed to be confirmed by EEG.

For PNES, clinical manifestations (e.g., shaking or unresponsiveness) in the absence of EEG abnormalities was used as diagnostic criterion.

For Parkinson’s disease, the Queens Square Brain Bank criteria were used diagnostic criteria (Lees A. J., Hardy, J. & Revesz, T., (2009). Parkinson’s disease, *The Lancet*, 373(9680), 2055-2066).

1. **Were study participants recruited in an appropriate way?**

Sampling has to be clearly stated. Reporting on all data (e.g., consecutive referrals) from a good census will identify everybody and is considered appropriate.

1. **Was the sample size adequate?**

Sample size was calculated using this formula: *n* = Z^2^ *P*(1-*P*)/*d*^2^, where *n* = sample size, Z = statistic for 95% level of confidence (1.96), *P* = expectation prevalence of PVT failure (15%, based upon Martin and Schroeder, 2021)), and *d* = precision (5%). This resulted in a minimal sample size of 196 subjects that was administered a PVT (per subgroup, in case results are displayed per subgroup).

1. **Were the study subjects and setting described in detail?**

Since PVT scores are potentially influenced by external gain incentives and language (-proficiency), these clinical variables are considered relevant in describing a target population

1. **Was there appropriate statistical analysis?**

The numerator (i.e., number subjects that failed a PVT) and denominator (i.e., total sample size of subjects provided a PVT) should be clearly reported.

*[Note: This is item 8 of the original format]*

1. **Was the response rate adequate, and if not, was the response rate managed appropriately?**

We defined response rate as the number of subjects that were initially described as eligible for inclusion in the study versus the final number of subjects in analyses. In case the initial sample was not fully included in the neuropsychological assessment beyond factors that clearly hamper cognitive functioning (e.g., postictal discharge), the dropouts must be compared with the included sample on relevant variables for PVT moderation (level of education, language-proficiency, and external gain incentives). In case there are no group-differences, the response rate was deemed appropriate.

*[Note: In this item, items 5 and 9 of the original format are combined]*
