# Supplementary material for: Performance Validity Test Failure in the Clinical Population: A Systematic Review and Meta-Analysis of Prevalence Rates
Source: Neuropsychol Rev. 2023 Mar 6;34(1):299–319. doi: 10.1007/s11065-023-09582-7 (PMC10920461; doi:10.1007/s11065-023-09582-7)
Supplement: Supplementary file 4 — Supplementary file4 (DOCX 20 KB) [file 11065_2023_9582_MOESM4_ESM.docx]

**Online Resource 4:**

**Table 3.**

*Study Quality Using the Joanna Briggs Institute’s critical Appraisal Checklist for Studies Reporting Prevalence Data*

| **Question** | **Cragar, 2006** | **Czornik, 2021** | **Dandachi-Fitzgerald, 2020** | **Davis, 2014** | **Deloria, 2021** | **Dodrill, 2008** | **Domen, 2020** | **Donders, 2011** | **Dorociak, 2018** | **Drane, 2006** | **Echstaedt, 2014** | **Erdodi, 2018** | **Galioto, 2020** | **Gorissen, 2005** | **Grote, 2000** | **Haber, 2006** | **Haggerty, 2007** | **Harrison, 2020** | **Harrison, 2021** | **Hoskins, 2010** | **Jeannette, 2021** | **Keary, 2013** | **Krishnan, 2011** | **Leppma, 2018** |
| --- | --- | --- | --- | --- | --- | --- | --- | --- | --- | --- | --- | --- | --- | --- | --- | --- | --- | --- | --- | --- | --- | --- | --- | --- |
| 1. Was the sample frame appropriate to address the target population? | Y | Y | Y | Y | Y | Y | Y | Y | Y | Y | Y | Y | N | Y | Y | Y | Y | Y | Y | Y | Y | N | Y | Y |
| 2. Were study participants sampled in an appropriate way? | Y | U | U | Y | N | Y | N | Y | Y | Y | N | N | N | N | N | Y | Y | Y | Y | Y | N | Y | Y | N |
| 3. Was the sample size adequate? | N | N | N | N | N | N | N | N | N | N | N | N | N | N | N | N | Y | Y | Y | N | N | Y | N | Y |
| 4. Were the study subjects and the setting described in detail? | N | N | Y | Y | N | N | Y | Y | N | N | Y | N | N | N | N | N | N | N | N | N | Y | N | N | N |
| 5. Was there appropriate statistical analysis? | Y | Y | N | N | N | Y | Y | Y | Y | Y | Y | Y | Y | Y | Y | Y | Y | Y | Y | Y | Y | Y | Y | Y |
| 6. Was the response rate adequate, and if not, was the low response rate managed appropriately? | Y | N | N | N | N | Y | N | Y | Y | Y | Y | Y | Y | Y | Y | Y | Y | Y | N | Y | Y | Y | Y | Y |

| **Question** | **Locke, 2008** | **Loring, 2007** | **Loring, 2005** | **Marshall, 2016** | **Martins, 2010** | **Merten, 2007** | **Meyers, 2014** | **Moore, 2005** | **Neale, 2020** | **Rees, 2001** | **Resch, 2021** | **Rhoads, 2021^a^** | **Rhoads, 2021^b^** | **Sabelli, 2021** | **Schroeder, 2019** | **Sharland, 2018** | **Sieck, 2013** | **Silverberg, 2017** | **Techner, 2004** | **Vilar-Lopez, 2021** | **Walter, 2014** | **Wodushek, 2021** | **Williamson, 2012** |
| --- | --- | --- | --- | --- | --- | --- | --- | --- | --- | --- | --- | --- | --- | --- | --- | --- | --- | --- | --- | --- | --- | --- | --- |
| 1. Was the sample frame appropriate to address the target population? | Y | N | N | Y | N | Y | Y | Y | Y | Y | Y | Y | Y | Y | Y | Y | Y | Y | Y | Y | Y | U | Y |
| 2. Were study participants sampled in an appropriate way? | Y | N | N | Y | U | U | U | Y | N | N | N | N | N | N | N | Y | U | Y | Y | N | N | N | Y |
| 3. Was the sample size adequate? | N | N | N | N | N | N | Y | N | N | N | N | N | N | Y | N | Y | N | N | N | N | N | N | N |
| 4. Were the study subjects and the setting described in detail? | N | N | N | N | Y | Y | N | N | N | N | N | Y | Y | N | N | Y | N | Y | N | Y | N | Y | Y |
| 5. Was there appropriate statistical analysis? | Y | Y | Y | N | Y | Y | Y | Y | Y | Y | Y | Y | N | Y | Y | N | Y | Y | Y | Y | Y | Y | Y |
| 6. Was the response rate adequate, and if not, was the low response rate managed appropriately? | Y | Y | Y | U | Y | Y | Y | Y | Y | Y | U | Y | N | Y | Y | Y | Y | N | Y | Y | Y | N | Y |

*Note:* N = No; U = Unclear; Y = Yes; item 5 = item 8 of the original format; item 6 = item 9 + item 5 of the original format.
