# Supplementary material for: Performance Validity Test Failure in the Clinical Population: A Systematic Review and Meta-Analysis of Prevalence Rates
Source: Neuropsychol Rev. 2023 Mar 6;34(1):299–319. doi: 10.1007/s11065-023-09582-7 (PMC10920461; doi:10.1007/s11065-023-09582-7)
Supplement: Supplementary file 5 — Supplementary file5 (DOCX 19 KB) [file 11065_2023_9582_MOESM5_ESM.docx]

**Online Resource 5:**

**Results of publication bias analyses**

*Figure 1. DOI plot and LKF-index*
